# Supply Chain Risk Diffusion in Partially Mapping Double-Layer Hypernetworks

**DOI:** 10.3390/e25050747

**Published:** 2023-05-02

**Authors:** Ping Yu, Zhiping Wang, Ya’nan Sun, Peiwen Wang

**Affiliations:** 1 School of Science, Dalian Maritime University, Dalian 116026, China; yp_20@dlmu.edu.cn (P.Y.); sunyanan0727@dlmu.edu.cn (Y.S.); 2 School of Maritime Economics and Management, Dalian Maritime University, Dalian 116026, China

**Keywords:** hypernetwork, partial mapping, dynamic evolution, supply chain risk, epidemic model

## Abstract

The impact of COVID-19 is global, and uncertain information will affect product quality and worker efficiency in the complex supply chain network, thus bringing risks. Aiming at individual heterogeneity, a partial mapping double-layer hypernetwork model is constructed to study the supply chain risk diffusion under uncertain information. Here, we explore the risk diffusion dynamics, drawing on epidemiology, and establish an SPIR (Susceptible–Potential–Infected–Recovered) model to simulate the risk diffusion process. The node represents the enterprise, and hyperedge represents the cooperation among enterprises. The microscopic Markov chain approach (MMCA) is used to prove the theory. Network dynamic evolution includes two removal strategies: (i) removing aging nodes; (ii) removing key nodes. Using Matlab to simulate the model, we found that it is more conducive to market stability to eliminate outdated enterprises than to control key enterprises during risk diffusion. The risk diffusion scale is related to interlayer mapping. Increasing the upper layer mapping rate to strengthen the efforts of official media to issue authoritative information will reduce the infected enterprise number. Reducing the lower layer mapping rate will reduce the misled enterprise number, thereby weakening the efficiency of risk infection. The model is helpful for understanding the risk diffusion characteristics and the importance of online information, and it has guiding significance for supply chain management.

## 1. Introduction

A supply chain consists of manufacturers, distributors, retailers, consumers, and so on. Products, funds, and information flow through the supply chain. To become bigger and stronger, enterprises will integrate into a larger economic environment, which has led to a shift in the supply chain from a traditional chain structure to a supply chain network structure. Enterprises on the supply chain network are interdependent, and any problem with one enterprise may affect other cooperating enterprises. In April 2020, COVID-19 caused serious damage to the world. According to data released by the World Health Organization, the global cumulative number of COVID-19 cases reached 3,018,952, and there were 2,934,583 confirmed cases outside China. Factory productivity declined, the household consumption index fell, and the real economy slumped significantly. Various risks brought by the epidemic have affected the increasingly complex supply chain network [1]. The risk diffusion process on the supply chain network is an extremely complex phenomenon, and its outbreak is often accompanied by the emergence of unconfirmed information. Social software such as Microblog, Forum, YouTube, and so on have accelerated the spread of uncertain information during the epidemic and intensified the risk diffusion. The official media guides the public’s cognition and is conducive to market stability [2]. In fact, not all enterprises have online social accounts that obtain uncertain information, and not all accounts follow the official media. The heterogeneity of individuals determines that the partially mapped double-layer hypernetwork is more suitable for studying the risk diffusion process under uncertain information. It helps managers realize the importance of Internet information management and is an important part of coupling dynamics research.

Research on supply chain risk is crucial to financial risk management [3,4]. There is a need to study the performance indicators and characteristics of the supply chain and develop a conceptual model for identifying supply chain risks [5], design an intuitionistic fuzzy two-stage supply chain network considering multi-mode demand and multi-mode transportation [6], and utilize machine learning to optimize supply chain management and maximize benefits on block-chained big data [7]. In addition to the main supply chain research mentioned above, the study of supply chain risk diffusion behavior referencing epidemic models is also a hot topic. In 1927, Kermark and McKendrick first constructed the SIR (Susceptible–Infected–Recovered) model to study the spreading scale of the London Black Death [8]. After that, considering that individuals will be susceptible again, the threshold theory of the SIS (Susceptible–Infected–Susceptible) model was proposed [9], which laid the foundation for future dynamics research [10]. The epidemic model is suitable for describing the risk diffusion behavior with contagiousness in the supply chain network. Wang et al. [11] used the SIR model to discuss the impact of multiple drivers including network heterogeneity on supply chain risk diffusion. Liang et al. [12] proved that there is a significant relationship between network structure and risk transmission and supply network health.

The above research found that the propagation dynamics model is mostly based on the theoretical knowledge of complex networks represented by graphs [3,11,12]. As a complex system, the supply chain has the characteristics of complexity, dynamics, and coordination. It is not the binary relationship that ordinary graphs express. At this time, it is particularly important to use nested mathematical theoretical tools to express multiple relationships. The hypernetwork was proposed by Sheff in 1985 [13]. It has the characteristics of multi-layer, multi-level, multi-dimensional, multi-attribute, and so on. Later, Estrada et al. [14] used hypergraphs to represent complex networks, extending the concepts of subgraph centrality and clustering. Hypergraphs have been applied in various fields. Suo et al. studied the dynamic process and macro behavior of the supply chain system with the evolutionary hypergraph theory [15]. Nodes represent individuals and hyperedges represent the relationship among individuals. Yin et al. described the non-uniform evolution of social networks [16] and obtained the explicit analytical expression of hyperdegree distribution. Hypernetworks are also used for risk dynamics research to explore the formation and development process of risk [17].

However, risk diffusion in supply chain networks is influenced by multiple factors. It is not comprehensive to capture the risk diffusion behavior of each node by building an isolated network [11,12]. Mukunda et al. [18] constructs a two-layer supply chain model under dynamic demand to optimize supply chain profits. Huo et al. [19] constructed multiple networks to analyze the impact of herd psychology and risk preference on risk propagation under warning information. To mitigate the damage caused by risk, it is reasonable to establish a risk transmission model with an information transmission effect [20]. Clear information will eliminate uncertainty and curb risk diffusion. Establishing a two-layer dynamic model and discussing the detailed process characteristics of the multiplexing network are of great significance [21,22]. Based on the above reasons, we deeply discuss the interaction between uncertain information and supply chain risk diffusion in partially mapping double-layer hypernetworks with the help of the SEIR epidemic model. The main contributions are as follows:

- Based on the partially mapping double-layer hypernetwork model, the randomness of the mapping between layers is proposed, and the interaction between supply chain risk and uncertain information is described.
- Two removal strategies are proposed to describe the network dynamic evolution process. The contribution of control means to market stability is analyzed.
- The supply chain risk diffusion process in a partially mapping double-layer hypernetwork with MATLAB is simulated, the effectiveness of MMCA is proved, and the risk diffusion trend under two strategies and dynamic evolution parameters is grasped.

The rest of the article is as follows. Section 2 describes the partially mapping double-layer hypernetwork structure and explains the applicability of the UBU-SPIR compartment model. Section 3 makes a detailed theoretical analysis of the risk diffusion process through MMCA. Section 4 uses MATLAB to carry out the numerical simulation, test the influence of parameters, and verify the correctness of the theoretical analysis. Finally, we sum up and prospect the risk diffusion dynamics in Section 5.

## 2. Model Description

In the complex supply chain network, risk diffusion is not an independent event. It will be affected by instant messages [23]. Correct information will improve vigilance [19], while uncertain information has the characteristics of wide coverage, strong communication power, and low communication cost, which will affect the public psychology and knowledge system [24] and give incorrect guidance to people’s activities. Therefore, it is in line with the actual need to use a hypernetwork to abstract the supply chain network system and analyze the risk diffusion process under the influence of uncertain information.

### 2.1. Hypernetwork Model

Hypernetworks are represented by hypergraphs. Suppose the supply chain network has n enterprises. There are complex cooperative relationships among enterprises. The hypergraph $H=\left\{V,E^{h}\right\}$ represents the supply chain network, the node set $V=\left\{v_{1},v_{2},\cdots ,v_{n}\right\}$ represents all enterprises in the supply chain network, and the node $v_{i}$ represents enterprises i. The hyperedge $E_{i}=\left\{v_{i1},v_{i2},\cdots ,v_{ig_{i}}\right\}\left(v_{iy}\in V,y=1,2,\cdots g_{i}\right)$ represents the cooperation formed by some enterprises, and the hyperedge set $E^{h}=\left\{E_{1},E_{2},\cdots ,E_{m}\right\}$ represents all cooperation in the supply chain network. Based on the hypernetwork theory, this paper discusses the interaction between uncertain information on virtual social networks and risk diffusion on supply chain networks. The hypernetwork representation of virtual social networks is similar [16]. In virtual social networks, the node represents the enterprise, and the hyperedge represents information exchanges that accompany cooperation between enterprises. Assume that the dynamic evolution of virtual social networks and supply chain networks is consistent, and some hyperedges added randomly represent the communication between non-cooperative enterprises. To simplify the model, assume the nodes in the double-layer network correspond to each other. However, only some enterprises have social accounts, and only some accounts follow official media, so only some nodes have mapping. In this paper, the vector $L=\left\{l_{1},l_{2},\cdots ,l_{N}\right\}$ represents the mapping between nodes in virtual social networks and nodes in supply chain networks, used to describe whether nodes in the supply chain networks have social accounts for information exchange in the virtual social networks. Introduce the official media O to suppress the uncertain information circulating in virtual social networks; the vector $L^{\prime }=\left\{l_{1}{}^{\prime },l_{2}{}^{\prime },\cdots ,l_{N}{}^{\prime }\right\}$ represents the mapping between the official media and each node in the virtual social network, used to describe whether nodes in the virtual social network follow official media accounts.

The market environment is changing rapidly, and commercial orders change frequently. The supply chain network continues to develop and expand with the growth of emerging enterprises, the increase in cooperation projects, and the withdrawal of some enterprises from the initial small-scale development to regionalization and even globalization. Assume that the rate of nodes entering and exiting the supply chain network is $\lambda_{1}$ and $\lambda_{2}\left(\lambda_{1}>\lambda_{2}\right)$, respectively, following the Poisson process. The number of nodes in each batch is fixed. The evolution behavior of the supply chain network gradually has certain regularity over time. Next, we describe the dynamic evolution process of the supply chain network with the knowledge of hypernetwork theory:

1. Enterprises in the supply chain network establish new cooperative relations.

Add m hyperedges with probability p. First, select a node randomly in the current supply chain network, and then form a new hyperedge with a nodes selected through the hyperdegree preferential attachment mechanism [25]. This process is repeated m times, corresponding to m new cooperation in the supply chain network.

2. When the contract expires, some enterprises no longer renew the contract with former partners but cooperate with new partners.

Rewire m hyperedges with probability q. First, select node i randomly in the current network to delete one of its hyperedges $e_{i}$, and then select node a as in Process 1 to form a new hyperedge $\tilde{e_{i}}$. This process is repeated m times, corresponding to the end of m old cooperation and the generation of m new cooperation in the supply chain network.

3. New enterprises enter, and some enterprises withdraw from the market.

Add $m_{2}$ nodes and remove $m_{1}$ nodes with probability r. Different strategies lead to different removal rules. The old node in the supply chain network is more vulnerable to risks than others because of its outdated technology and weak resistance to risks. If key enterprises are infected by risk, it will cause multiple nodes to be affected by risk. Therefore, we consider removing aging nodes according to the appearance order of the node or removing key nodes according to the hyperdegree to inhibit the risk diffusion.

**Figure 1:**
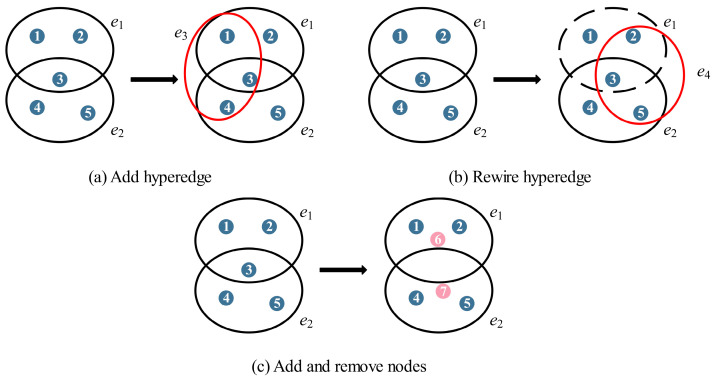
Dynamic evolution of the network. One of the three processes will occur at t+1. (**a**) Add hyperedge $e_{3}$ containing nodes 1, 3, and 4; (**b**) Randomly select a hyperedge $e_{1}$ containing node 3 to remove, and then form a hyperedge $e_{4}$ with nodes 2 and 5; (**c**) Remove node 3 and add nodes 6 and 7 in the system. Blue node represents the old node, pink node represents the new node.

Figure 1 is an example of the dynamic evolution of the supply chain network. Table 1 shows the parameters related to the model.

### 2.2. UBU-SPIR Compartment Model

**Figure 2:**
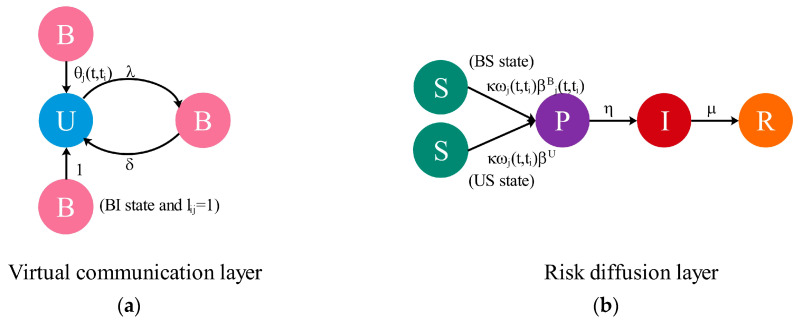
State transition of the nodes in each layer. Left panel (**a**) shows the node state transition during the dissemination of uncertain information. Right panel (**b**) shows the node state transition in the process of supply chain risk diffusion.

In the process of supply chain risk diffusion, uncertain information will accelerate the diffusion of risk, while the official media restricts the spread of uncertain information. This paper constructs a partially mapped double-layer hypernetwork; the upper layer is the virtual social network that describes the process of uncertain information dissemination, and the lower layer is a supply chain network that spreads risks through cooperation. In the past research on information dissemination, nodes only considered two states [26]. Therefore, as shown in Figure 2a, on the virtual communication layer, the enterprise transitions between two states through the UBU (Unbelieve–Believe–Unbelieve) model. λ indicates the probability that the U-state enterprise believes information after contacting the B-state neighbor. δ indicates the probability that the B-state enterprises no longer believe. In addition to the interaction between enterprises, the official media will release authoritative information, which will prevent the spread of uncertain information. $\theta_{j}\left(t,t_{i}\right)$ indicates the probability that the jth B-state enterprise in the ith batch no longer believes under the influence of the official media.

(1) $$\theta_{j}\left(t,t_{i}\right)=\left\{ \begin{array}{l} \tau \;,\;l_{ij}{}^{\prime }=1\\ 0\;,\;l_{ij}{}^{\prime }=0 \end{array}\right.,$$

where τ indicates the probability that enterprises that follow the official media no longer believe after receiving the authoritative information. $l_{ij}{}^{\prime }$ denotes the mapping between the jth node of the ith batch in the virtual communication layer and official media. The risks on the supply chain network are contagious. If an enterprise’s demand for raw materials decreases, it will impact the suppliers at the front end of the supply chain. If the supplier’s raw material is insufficient, the backend of the supply chain will suffer production damage. Any problem in any link of the supply chain will interfere with other cooperative enterprises, leading to risk diffusion. On the risk diffusion layer. Due to the existence of potential risks, the SPIR (Susceptible–Potential–Infected–Recovered) model is used to simulate the risk diffusion in the supply chain network. The hyperdegree is the number of hyperedges contained in a node. Enterprises with a greater hyperdegree $h_{j}(t,t_{i})$ are more likely to be exposed to risks. In Figure 2b, susceptible enterprises contact with infected enterprises or enterprises with potential risk with probability $\omega_{j}(t,t_{i})$ and then convert to the P-state with probability β. η indicates the probability of being completely infected by the risk after the potential risk is serious. Infected enterprises recover at recovery rate μ and no longer involve such risks.

(2) $$\omega_{j}(t,t_{i})=\frac{h_{j}(t,t_{i})}{Max\left(h(t)\right)},$$

where $h_{j}(t,t_{i})$ indicates the hyperdegree of the jth node in the ith batch. Max(h(t)) indicates the maximum hyperdegree at time t.

The risk diffusion on the supply chain network and the transmission of uncertain information on the virtual social network are interactive processes. If there is a mapping between the two layers ($l_{ij}=1$), the infected enterprise in the risk diffusion layer will immediately realize the error of the uncertain information on the virtual communication layer, and then it will be transformed into a U-state. No mapping has no effect. For susceptible enterprises at the risk diffusion layer, the probability of enterprises misled by information being infected must be greater than that of Unbelieve-enterprises being infected. Hence, enterprises that believe in uncertain information change from the S-state to the P-state with probability $\beta_{j}{}^{B}\left(t,t_{i}\right)=\Gamma_{j}\left(t,t_{i}\right)\beta$, $\Gamma_{j}\left(t,t_{i}\right)$ is an inter-layer reinforcement factor, and $\beta^{U}=\beta$ denotes the probability that Unbelieve-enterprises change from the S-state to the P-state.

(3) $$\Gamma_{j}\left(t,t_{i}\right)=\left\{ \begin{array}{l} \gamma \;,\;l_{ij}=1\\ 1\;,\;l_{ij}=0 \end{array}\right.,$$

$\Gamma_{j}\left(t,t_{i}\right)$ is related to interlayer mapping. γ(γ>1) indicates the inter-layer enhancement factor. $l_{ij}$ indicates the mapping between the two layers of the jth node in the ith batch. $\beta_{1}=\gamma \cdot \beta$ indicates the probability that the B-state enterprise changes from the S-state to the P-state when there is a mapping between the two layers. Considering the UBU-SPIR compartment model and inter-layer influence, the enterprise in the model will be in one of eight states: the US state (Unbelieve and Susceptible), UP state (Unbelieve and Potential), UI state (Unbelieve and Infected), UR state (Unbelieve and Recovered), BS state (Believe and Susceptible), BP state (Believe and Potential), BI state (Believe and Infected), or BR state (Believe and Recovered).

### 2.3. Dynamic Evolution Steps of the Model

Under the influence of uncertain information, the supply chain risk diffusion model on the partially mapping double-layer hypernetwork first undergoes network dynamic evolution at each time step t and then undergoes dynamic propagation. In the process of dynamic propagation, three dynamic processes occur at the same time: the dissemination of uncertain information (UBU), the release of authoritative information from official media, and the diffusion of risk (SPIR). The goal of this paper is to obtain the proportion of recovered enterprises in a stable state of the system. The dynamic evolution steps of the entire model are as follows:

**Step 1:** Network initialization. The initial number of nodes is 16, and an initial network of 1000 nodes is formed through the network dynamic evolution Process 1–3 in Section 2.1. The upper is the virtual communication layer, the lower is the risk diffusion layer. O represents the official media. Add 30 hyperedges randomly in the virtual communication layer to represent the information exchange between non-cooperative enterprises.

**Step 2:** The dynamic evolution of the double-layer hypernetwork. At each time step t, the dynamic evolution Process 1–3 in Section 2.1 randomly occur in the supply chain network and virtual social network with the probabilities p, q, and r.

**Step 3:** Node state transition. At each time step t, nodes in the double-layer network update states according to Figure 2. The nodes of the virtual communication layer and the risk diffusion layer interact through partial mapping.

**Figure 3:**
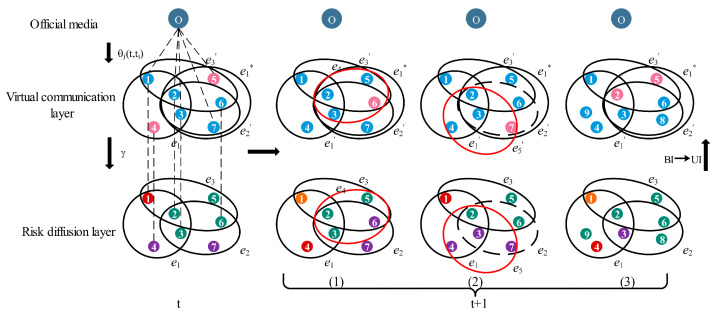
The risk diffusion process in the partially mapping double-layer hypernetwork. The virtual communication layer contains two states: Unbelieve (blue) and Believe (pink). The risk diffusion layer contains four states: Susceptible (green), Potential (purple), Infected (red), and Recovered (orange). Process (1)–(3) correspond to the three processes (a)–(c) in Figure 1. The red link represents the new hyperedge, and the dotted line represents the deleted hyperedge. $e_{1}^{*}$ denotes communication between enterprises without cooperation. Numbers represent serial numbers of a node or a hyperedge.

The supply chain risk diffusion process on the partially mapping double-layer hypernetwork continues to cycle from step 2 to step 3 until the number of nodes in each state is stable, i.e., the system reaches the stable state. The dynamic evolution process of the entire model is shown in Figure 3.

## 3. Theoretical Analysis

As is known to all, the risk diffusion scale is related to the network structure [11]. Different node removal strategies lead to different network evolution processes, which will be beneficial to inhibiting risks. To study the impact of uncertain information, we derive the key parameters under different removal strategies and then use MMCA to obtain the proportion of enterprise in various states when the system is stable.

### 3.1. Remove the Aging Node

If the removal of Process 3 is determined by the node life [27], $h_{j}(t,t_{i})$ affected by Process 1–3 in Section 2.1 will meet the following three dynamic equations:

(4) $$\frac{\partial h_{j}(t,t_{i})}{\partial t}=pm\left(\lambda_{1}-\lambda_{2}\right)\left[\frac{1}{N(t)}+a\frac{h_{j}(t,t_{i})+1}{\sum_{ij}\left(h_{j}(t,t_{i})+1\right)}\right],$$

(5) $$\frac{\partial h_{j}(t,t_{i})}{\partial t}=qm\left(\lambda_{1}-\lambda_{2}\right)\left[-\frac{1}{N(t)}+a\frac{h_{j}(t,t_{i})+1}{\sum_{ij}\left(h_{j}(t,t_{i})+1\right)}\right],$$

(6) $$\frac{\partial h_{j}(t,t_{i})}{\partial t}=-rm_{1}\lambda_{2}\frac{t_{ij}^{-1}}{\sum_{ij}t_{ij}^{-1}}h_{j}(t,t_{i}),$$

where $t_{ij}$ represents the time when the jth node in the ith batch enters the system.

In fact, in a unit time step, three steps occur simultaneously. Thus, the hyperdegree $h_{j}(t,t_{i})$ change should include three dynamic processes. Combined with Equations (4)–(6), we can obtain:

(7) $$\frac{\partial h_{j}(t,t_{i})}{\partial t}=\frac{m\left(\lambda_{1}-\lambda_{2}\right)}{N(t)}\left(p-q\right)+a\frac{h_{j}(t,t_{i})+1}{\sum_{ij}\left(h_{j}(t,t_{i})+1\right)}m\left(\lambda_{1}-\lambda_{2}\right)\left(p+q\right)-r\lambda_{2}m_{1}\frac{t_{ij}^{-1}}{\sum_{ij}t_{ij}^{-1}}h_{j}(t,t_{i}).$$

When t→∞, $N(t)\approx \left(\lambda_{1}-\lambda_{2}\right)t$, and $\sum_{ij}\left(h_{j}(t,t_{i})+1\right)\approx \left[pm(1+a)+m_{2}-rm_{1}\right]E\left[N(t)\right]$. Let $A=\underset{t\to \infty }{\lim}\frac{\sum_{ij}t_{ij}^{-1}}{N(t)}$ and $G=\frac{A}{m_{1}}$; Equation (7) can be written as:

(8) $$\frac{\partial h_{j}(t,t_{i})}{\partial t}=\frac{1}{t}\left\{m\left(p-q\right)+\left[\frac{am\left(p+q\right)}{pm\left(1+a\right)+m_{2}-rm_{1}}-\frac{r\lambda_{2}t_{ij}^{-1}}{G\left(\lambda_{1}-\lambda_{2}\right)}\right]h_{j}(t,t_{i})+\frac{am\left(p+q\right)}{pm(1+a)+m_{2}-rm_{1}}\right\}.$$

From the initial conditions, we know that the jth node in the ith batch reaches the system at $t_{i}$; thus, $h_{j}(t_{i},t_{i})=r$. Let $Q=\frac{am\left(p+q\right)}{pm(1+a)+m_{2}-rm_{1}}$, J=m(p−q), and $K=\frac{am\left(p+q\right)}{pm(1+a)+m_{2}-rm_{1}}-\frac{r\lambda_{2}t_{ij}^{-1}}{G\left(\lambda_{1}-\lambda_{2}\right)}.$. Solving Equation (8), we obtain

(9) $$h_{j}(t,t_{i})=\left(r+\frac{J+Q}{K}\right){\left(\frac{t}{t_{i}}\right)}^{K}-\frac{J+Q}{K}.$$

Therefore,

(10) $$\omega_{j}\left(t,t_{i}\right)=\frac{\left(r+\frac{J+Q}{K}\right){\left(\frac{t}{t_{i}}\right)}^{K}-\frac{J+Q}{K}}{Max\left(h(t)\right)}.$$

### 3.2. Remove the Key Node

If the Process 3 is to select key nodes for removal, for the three equations that $h_{j}(t,t_{i})$ meets, the first two equations are the same as Equations (4) and (5), and the third equation is as follows:

(11) $$\frac{\partial h_{j}(t,t_{i})}{\partial t}=-rm_{1}\lambda_{2}\frac{h_{j}(t,t_{i})+1}{\sum_{ij}\left(h_{j}(t,t_{i})+1\right)}h_{j}(t,t_{i}).$$

Combining Equations (4), (5), and (11), Equation (12) is obtained:

(12) $$\frac{\partial h_{j}(t,t_{i})}{\partial t}=\frac{m\left(\lambda_{1}-\lambda_{2}\right)}{N(t)}\left(p-q\right)+m\left(\lambda_{1}-\lambda_{2}\right)a\frac{h_{j}(t,t_{i})+1}{\sum_{ij}\left(h_{j}(t,t_{i})+1\right)}\left(p+q\right)-rm_{1}\lambda_{2}\frac{h_{j}(t,t_{i})+1}{\sum_{ij}\left(h_{j}(t,t_{i})+1\right)}h_{j}(t,t_{i}).$$

When t→∞, $N(t)\approx \left(\lambda_{1}-\lambda_{2}\right)t$, and $\sum_{ij}\left(h_{j}(t,t_{i})+1\right)\approx \left[pm\left(a+1\right)+m_{2}-rm_{1}\right]E\left[N(t)\right]$. Therefore,

(13) $$\begin{array}{ll} \frac{\partial h_{j}(t,t_{i})}{\partial t} & =\frac{1}{t}\{m\left(p-q\right)+\frac{ma\left(\lambda_{1}-\lambda_{2}\right)\left(p+q\right)}{\left[pm\left(1+a\right)+m_{2}-rm_{1}\right]\left(\lambda_{1}-\lambda_{2}\right)}\left(h_{j}(t,t_{i})+1\right)\\ & -\frac{rm_{1}\lambda_{2}}{\left[pm(1+a)+m_{2}-rm_{1}\right]\left(\lambda_{1}-\lambda_{2}\right)}\left(h_{j}(t,t_{i})+1\right)h_{j}(t,t_{i})\}. \end{array}$$

Let $K^{\prime }=\frac{ma\left(\lambda_{1}-\lambda_{2}\right)\left(p+q\right)}{\left[pm\left(a+1\right)+m_{2}-rm_{1}\right]\left(\lambda_{1}-\lambda_{2}\right)}$, $Q^{\prime }=\frac{rm_{1}\lambda_{2}}{\left[pm\left(a+1\right)+m_{2}-rm_{1}\right]\left(\lambda_{1}-\lambda_{2}\right)}$, and $J^{\prime }=m\left(p-q\right)$. Then, integrate both sides of Equation (13) and finally obtain

(14) $$h_{j}\left(t,t_{i}\right)=\frac{K^{\prime }-Q^{\prime }}{2Q^{\prime }}-\frac{\sqrt{4Q^{\prime }\left(J^{\prime }+K^{\prime }\right)+{\left(Q^{\prime }-K^{\prime }\right)}^{2}}}{2Q^{\prime }}\cdot Pa.$$

$$Pa=\frac{2Q^{\prime }r+Q^{\prime }-K^{\prime }+\sqrt{4Q^{\prime }\left(J^{\prime }+K^{\prime }\right)+{\left(Q^{\prime }-K^{\prime }\right)}^{2}}+\left[2Q^{\prime }r+Q^{\prime }-K^{\prime }-\sqrt{4Q^{\prime }\left(J^{\prime }+K^{\prime }\right)+{\left(Q^{\prime }-K^{\prime }\right)}^{2}}\right]{\left(\frac{t}{t_{i}}\right)}^{-\sqrt{4Q^{\prime }\left(J^{\prime }+K^{\prime }\right)+{\left(Q^{\prime }-K^{\prime }\right)}^{2}}}}{2Q^{\prime }r+Q^{\prime }-K^{\prime }-\sqrt{4Q^{\prime }\left(J^{\prime }+K^{\prime }\right)+{\left(Q^{\prime }-K^{\prime }\right)}^{2}}{\left(\frac{t}{t_{i}}\right)}^{-\sqrt{4Q^{\prime }\left(J^{\prime }+K^{\prime }\right)+{\left(Q^{\prime }-K^{\prime }\right)}^{2}}}-\left[2Q^{\prime }r+Q^{\prime }-K^{\prime }+\sqrt{4Q^{\prime }\left(J^{\prime }+K^{\prime }\right)+{\left(Q^{\prime }-K^{\prime }\right)}^{2}}\right]}.$$

The probability of the jth node in the ith batch encountering the risk is obtained by Equations (2) and (14).

**Figure 4:**
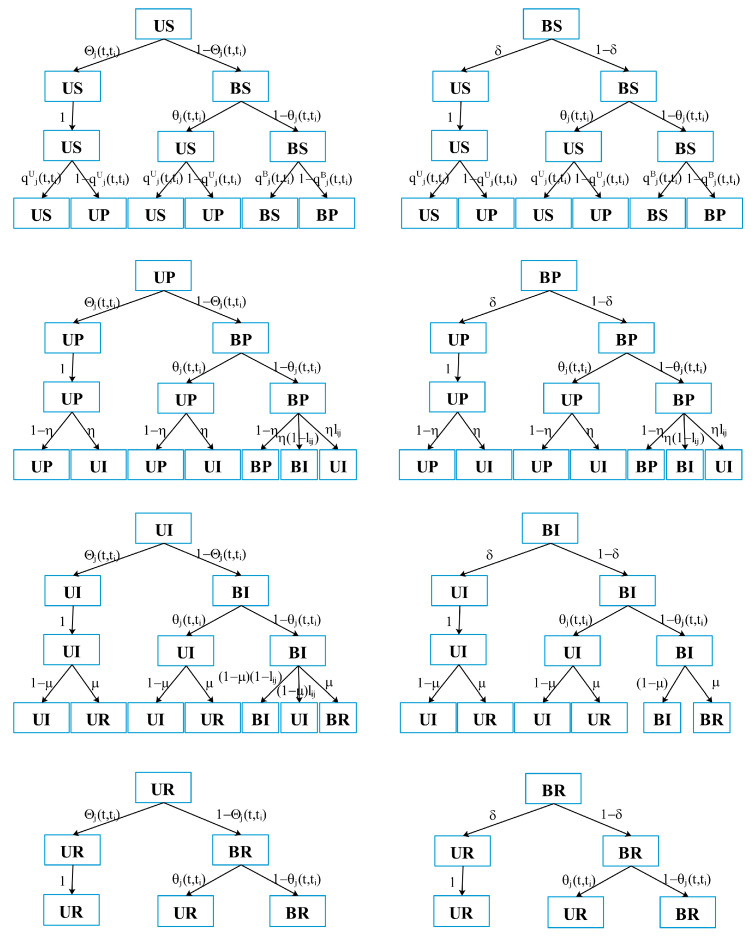
State transition probability tree.

At each time step t, the node is in one of eight states with the probabilities $P_{j}^{US}\left(t,t_{i}\right)$, $P_{j}^{UP}\left(t,t_{i}\right)$, $P_{j}^{UI}\left(t,t_{i}\right)$, $P_{j}^{UR}\left(t,t_{i}\right)$, $P_{j}^{BS}\left(t,t_{i}\right)$, $P_{j}^{BP}\left(t,t_{i}\right)$, $P_{j}^{BI}\left(t,t_{i}\right)$, and $P_{j}^{BR}\left(t,t_{i}\right)$, respectively, and meets the normalization conditions $P_{j}^{US}\left(t,t_{i}\right)+P_{j}^{UP}\left(t,t_{i}\right)+P_{j}^{UI}\left(t,t_{i}\right)+P_{j}^{UR}\left(t,t_{i}\right)$$+P_{j}^{BS}\left(t,t_{i}\right)+P_{j}^{BP}\left(t,t_{i}\right)+P_{j}^{BI}\left(t,t_{i}\right)+P_{j}^{BR}\left(t,t_{i}\right)\equiv 1$. In the risk diffusion layer, $n_{ij}$ represents the jth node in the ith batch. If there is a relationship between the nodes $n_{ij}$ and $n_{zf}$, then $c_{ijzf}=1$; otherwise, $c_{ijzf}=0$. So, in the virtual communication layer, $d_{ijzf}=1$ means there is a relationship between the nodes $n_{ij}$ and $n_{zf}$, and $d_{ijzf}=0$ means there is no relationship. Let $\Theta_{j}\left(t,t_{i}\right)$ indicate the probability that the U-state node does not receive uncertain information from neighbors, $q_{j}^{U}\left(t,t_{i}\right)$ indicate the probability that the U-state node is infected by risk, and $q_{j}^{B}\left(t,t_{i}\right)$ indicate the probability that the B-state node is infected by risk. Combined with Figure 2, the state transition probability tree of eight states is shown in Figure 4.

(15) $$\Theta_{j}\left(t,t_{i}\right)=\underset{z}{\Pi }\underset{f}{\Pi }\left[1-d_{ijzf}\lambda P_{f}^{B}\left(t,t_{z}\right)\right],$$

(16) $$q_{j}^{U}\left(t,t_{i}\right)=\underset{z}{\Pi }\underset{f}{\Pi }\left\{1-c_{ijzf}\left[P_{f}^{P}\left(t,t_{z}\right)+P_{f}^{I}\left(t,t_{z}\right)\right]\kappa \omega_{j}\left(t,t_{i}\right)\beta^{U}\right\},$$

(17) $$q_{j}^{B}\left(t,t_{i}\right)=\underset{z}{\Pi }\underset{f}{\Pi }\left\{1-c_{ijzf}\left[P_{f}^{P}\left(t,t_{z}\right)+P_{f}^{I}\left(t,t_{z}\right)\right]\kappa \omega_{j}\left(t,t_{i}\right)\beta_{j}^{B}\left(t,t_{i}\right)\right\},$$

where κ is the adjustment coefficient. $P_{f}^{B}\left(t,t_{z}\right)=P_{f}^{BS}\left(t,t_{z}\right)+P_{f}^{BP}\left(t,t_{z}\right)+P_{f}^{BI}\left(t,t_{z}\right)+P_{f}^{BR}\left(t,t_{z}\right)$, $P_{f}^{P}\left(t,t_{z}\right)=P_{f}^{UP}\left(t,t_{z}\right)+P_{f}^{BP}\left(t,t_{z}\right)$, $P_{f}^{I}\left(t,t_{z}\right)=P_{f}^{UI}\left(t,t_{z}\right)+P_{f}^{BI}\left(t,t_{z}\right)$.

MMCA has been used for dynamic research many times, including disease spreading and information dissemination [28,29,30]. It can track the node state in the supply chain risk diffusion process [19]. According to the state transition probability tree in Figure 4, the dynamic transition process of each node state in the model is written as:

(18) $$\begin{array}{ll} P_{j}^{US}\left(t+1,t_{i}\right) & =P_{j}^{US}\left(t,t_{i}\right)\left\{\Theta_{j}\left(t,t_{i}\right)q_{j}^{U}\left(t,t_{i}\right)+\left[1-\Theta_{j}\left(t,t_{i}\right)\right]\theta_{j}\left(t,t_{i}\right)q_{j}^{U}\left(t,t_{i}\right)\right\}\\ & +P_{j}^{BS}\left(t,t_{i}\right)\left[\delta q_{j}^{U}\left(t,t_{i}\right)+\left(1-\delta \right)\theta_{j}\left(t,t_{i}\right)q_{j}^{U}\left(t,t_{i}\right)\right] \end{array},$$

(19) $$\begin{array}{ll} P_{j}^{BS}\left(t+1,t_{i}\right) & =P_{j}^{US}\left(t,t_{i}\right)\left[1-\Theta_{j}\left(t,t_{i}\right)\right]\left[1-\theta_{j}\left(t,t_{i}\right)\right]q_{j}^{B}\left(t,t_{i}\right)\\ & +P_{j}^{BS}\left(t,t_{i}\right)\left(1-\delta \right)\left[1-\theta_{j}\left(t,t_{i}\right)\right]q_{j}^{B}\left(t,t_{i}\right) \end{array},$$

(20) $$\begin{array}{ll} P_{j}^{UP}\left(t+1,t_{i}\right) & =P_{j}^{US}\left(t,t_{i}\right)\left\{\Theta_{j}\left(t,t_{i}\right)\left[1-q_{j}^{U}\left(t,t_{i}\right)\right]+\left[1-\Theta_{j}\left(t,t_{i}\right)\right]\theta_{j}\left(t,t_{i}\right)\left[1-q_{j}^{U}\left(t,t_{i}\right)\right]\right\}\\ & +P_{j}^{BS}\left(t,t_{i}\right)\left\{\delta \left[1-q_{j}^{U}\left(t,t_{i}\right)\right]+\left(1-\delta \right)\theta_{j}\left(t,t_{i}\right)\left[1-q_{j}^{U}\left(t,t_{i}\right)\right]\right\}+P_{j}^{UP}\left(t,t_{i}\right)\\ & \left\{\Theta_{j}\left(t,t_{i}\right)\left(1-\eta \right)+\left[1-\Theta_{j}\left(t,t_{i}\right)\right]\theta_{j}\left(t,t_{i}\right)\left(1-\eta \right)\right\}+P_{j}^{BP}\left(t,t_{i}\right)[\delta \left(1-\eta \right)\\ & +\left(1-\delta \right)\theta_{j}\left(t,t_{i}\right)\left(1-\eta \right)] \end{array},$$

(21) $$\begin{array}{ll} P_{j}^{BP}\left(t+1,t_{i}\right) & =P_{j}^{US}\left(t,t_{i}\right)\left[1-\Theta_{j}\left(t,t_{i}\right)\right]\left[1-\theta_{j}\left(t,t_{i}\right)\right]\left[1-q_{j}^{B}\left(t,t_{i}\right)\right]+P_{j}^{BS}\left(t,t_{i}\right)\\ & \left(1-\delta \right)\left[1-\theta_{j}\left(t,t_{i}\right)\right]\left[1-q_{j}^{B}\left(t,t_{i}\right)\right]+P_{j}^{UP}\left(t,t_{i}\right)\left[1-\Theta_{j}\left(t,t_{i}\right)\right]\\ & \left[1-\theta_{j}\left(t,t_{i}\right)\right]\left(1-\eta \right)+P_{j}^{BP}\left(t,t_{i}\right)\left(1-\delta \right)\left[1-\theta_{j}\left(t,t_{i}\right)\right]\left(1-\eta \right) \end{array},$$

(22) $$\begin{array}{ll} P_{j}^{UI}\left(t+1,t_{i}\right) & =P_{j}^{UP}\left(t,t_{i}\right)\left\{\Theta_{j}\left(t,t_{i}\right)\eta +\left[1-\Theta_{j}\left(t,t_{i}\right)\right]\theta_{j}\left(t,t_{i}\right)\eta +\left[1-\Theta_{j}\left(t,t_{i}\right)\right]\left[1-\theta_{j}\left(t,t_{i}\right)\right]\eta l_{ij}\right\}\\ & +P_{j}^{BP}\left(t,t_{i}\right)\left\{\delta \eta +\left(1-\delta \right)\theta_{j}\left(t,t_{i}\right)\eta +\left(1-\delta \right)\left[1-\theta_{j}\left(t,t_{i}\right)\right]\eta l_{ij}\right\}+P_{j}^{UI}\left(t,t_{i}\right)\\ & \{\Theta_{j}\left(t,t_{i}\right)\left(1-\mu \right)+\left[1-\Theta_{j}\left(t,t_{i}\right)\right]\theta_{j}\left(t,t_{i}\right)\left(1-\mu \right)+\left[1-\Theta_{j}\left(t,t_{i}\right)\right]\left[1-\theta_{j}\left(t,t_{i}\right)\right]\\ & \left(1-\mu \right)l_{ij}\}+P_{j}^{BI}\left(t,t_{i}\right)\left[\delta \left(1-\mu \right)+\left(1-\delta \right)\theta_{j}\left(t,t_{i}\right)\left(1-\mu \right)\right] \end{array},$$

(23) $$\begin{array}{cc} P_{j}^{BI}\left(t+1,t_{i}\right) & =P_{j}^{UP}\left(t,t_{i}\right)\left[1-\Theta_{j}\left(t,t_{i}\right)\right]\left[1-\theta_{j}\left(t,t_{i}\right)\right]\eta \left(1-l_{ij}\right)+P_{j}^{BP}\left(t,t_{i}\right)\\ & \left(1-\delta \right)\left[1-\theta_{j}\left(t,t_{i}\right)\right]\eta \left(1-l_{ij}\right)+P_{j}^{UI}\left(t,t_{i}\right)\left[1-\Theta_{j}\left(t,t_{i}\right)\right]\\ & \left[1-\theta_{j}\left(t,t_{i}\right)\right]\left(1-\mu \right)\left(1-l_{ij}\right)+P_{j}^{BI}\left(t,t_{i}\right)\left(1-\delta \right)\left[1-\theta_{j}\left(t,t_{i}\right)\right]\left(1-\mu \right) \end{array},$$

(24) $$\begin{array}{ll} P_{j}^{UR}\left(t+1,t_{i}\right) & =P_{j}^{UI}\left(t,t_{i}\right)\left\{\Theta_{j}\left(t,t_{i}\right)\mu +\left[1-\Theta_{j}\left(t,t_{i}\right)\right]\theta_{j}\left(t,t_{i}\right)\mu \right\}+P_{j}^{BI}\left(t,t_{i}\right)[\delta \mu +\left(1-\delta \right)\theta_{j}\left(t,t_{i}\right)\\ & \mu ]+P_{j}^{UR}\left(t,t_{i}\right)\left\{\Theta_{j}\left(t,t_{i}\right)+\left[1-\Theta_{j}\left(t,t_{i}\right)\right]\theta_{j}\left(t,t_{i}\right)\right\}+P_{j}^{BR}\left(t,t_{i}\right)\left[\delta +\left(1-\delta \right)\theta_{j}\left(t,t_{i}\right)\right] \end{array},$$

(25) $$\begin{array}{ll} P_{j}^{BR}\left(t+1,t_{i}\right) & =P_{j}^{UI}\left(t,t_{i}\right)\left[1-\Theta_{j}\left(t,t_{i}\right)\right]\left[1-\theta_{j}\left(t,t_{i}\right)\right]\mu +P_{j}^{BI}\left(t,t_{i}\right)\left(1-\delta \right)\left[1-\theta_{j}\left(t,t_{i}\right)\right]\mu \\ & +P_{j}^{UR}\left(t,t_{i}\right)\left[1-\Theta_{j}\left(t,t_{i}\right)\right]\left[1-\theta_{j}\left(t,t_{i}\right)\right]+P_{j}^{BR}\left(t,t_{i}\right)\left(1-\delta \right)\left[1-\theta_{j}\left(t,t_{i}\right)\right] \end{array}.$$

According to the Markov Chain Approach, node state transitions occur over time. The state probability of any node at any time is obtained.

(26) $$P_{j}^{R}\left(t+1,t_{i}\right)=P_{j}^{UR}\left(t+1,t_{i}\right)+P_{j}^{BR}\left(t+1,t_{i}\right)=\mu P_{j}^{I}\left(t,t_{i}\right)+P_{j}^{R}\left(t,t_{i}\right),$$

(27) $$P_{j}^{I}\left(t+1,t_{i}\right)=P_{j}^{UI}\left(t+1,t_{i}\right)+P_{j}^{BI}\left(t+1,t_{i}\right)=\eta P_{j}^{P}\left(t,t_{i}\right)+\left(1-\mu \right)P_{j}^{I}\left(t,t_{i}\right).$$

The system tends to be stable when the time step is large enough. Thus, when t→∞, $P_{j}^{I}\left(t+1,t_{i}\right)\approx P_{j}^{I}\left(t,t_{i}\right)\approx P_{ij}^{I}$, and $P_{j}^{R}\left(t+1,t_{i}\right)\approx P_{j}^{R}\left(t,t_{i}\right)\approx P_{ij}^{R}$. $P_{j}^{R}\left(t+1,t_{i}\right)-P_{j}^{R}\left(t,t_{i}\right)<\varepsilon$. Equation (26) can be written as

(28) $$\mu P_{ij}^{I}<\varepsilon ,\;P_{ij}^{I}<\frac{\varepsilon }{\mu }.$$

This shows that the proportion of infected enterprises approaches 0 when the system is stable. According to Equation (27), we will obtain the probability under a stable state

(29) $$P_{ij}^{I}=\frac{\eta }{\mu }P_{ij}^{P}.$$

This paper considers the heterogeneity of individuals. Official media, virtual social networks, and supply chain networks are all partially mapped. Furthermore, we compared the removal of key nodes with aging nodes to find the best strategy for mitigating risk. In fact, if $L=L^{\prime }=\left\{1,1,\cdots ,1\right\}$, nodes exit according to the lifetime during the dynamic evolution of the network (Section 3.1). The model will degenerate into the risk diffusion model in Ref. [27].

## 4. Numerical Simulation

In the study of risk diffusion, the proportion of recovered enterprises $\rho^{R}$ in the stable state is an important indicator for judging the effect of risk mitigation. In this section, we will discuss the influence of model parameters on $\rho^{R}$ through Monte Carlo (MC) simulation and MMCA iterative calculation. In the iterative calculation of MMCA, $\rho^{R}=\frac{\sum_{ij}P_{ij}^{UR}+P_{ij}^{BR}}{N}\times 100\%$. In MC simulation, $\rho^{R}=\frac{N^{UR}+N^{BR}}{N}\times 100\%$. $P_{ij}^{UR}$ and $P_{ij}^{BR}$, respectively, represent the probability that the jth node in the ith batch is in the UR and BR states, while $N^{UR}$ and $N^{BR}$ represent the number of nodes in the UR and BR states, respectively. N represents the total number of nodes. The setting parameter of the initial network is a=5, m=2, $m_{1}=5$, $m_{2}=7$, p=0.3, and q=0.25. Consider the dynamic evolution of the network; let, p+q+r≡1. The upper layer mapping rate and the lower layer mapping rate are defined as $\psi =\frac{\sum_{ij}l_{ij}{}^{\prime }}{N}\times 100\%$ and $\phi =\frac{\sum_{ij}l_{ij}}{N}\times 100\%$, respectively, where $l_{ij}{}^{\prime }\in L^{\prime }$, and $l_{ij}\in L$. When the simulation starts, the mapping is randomly generated, and the mapping rate is 80%. Furthermore, 10 nodes are randomly selected to set to the P-state. B-state nodes are generated in the same way.

### 4.1. Removal of Aging Nodes

**Figure 5:**
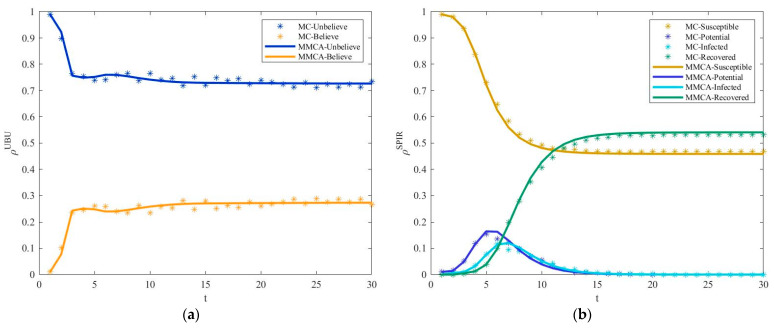
Under the strategy of removing aging nodes. (**a**) The fraction of U-state and B-state enterprises as a function with time step t. (**b**) The fraction of S-state, P-state, I-state, and R-state enterprises as a function with time step t. Parameters are set as follows: p=0.5, q=0.45, β=0.5, η=0.6, μ=0.8, λ=0.5, δ=0.3, τ=0.8, γ=2, and κ=2.

First, to verify the effectiveness of MMCA in characterizing the supply chain risk diffusion in the partially mapping double-layer hypernetwork under the strategy of removing aging nodes, Figure 5 describes the change in the proportion of enterprises in each state with time t. Then, the average relative error between MMCA and MC simulation is calculated using the formula $\left|\rho^{MC}-\rho^{MMCA}\right|/\rho^{MC}$. The result shows that the point plot and line plot basically overlap. The relative error of the proportion of enterprises in the U-, B-, S-, and R-states is 1.42%, 4.58%, 1.81%, and 4.55%, respectively. Hence, MMCA can be used to simulate the risk diffusion process of the supply chain in a partially mapping double-layer hypernetwork.

**Figure 6:**
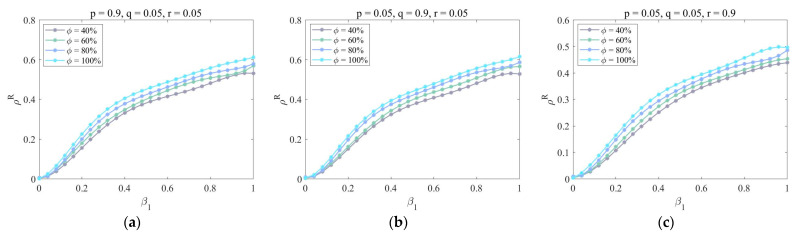
The impact of dynamic parameters (p, q, r) and the lower layer mapping rate ϕ under the strategy of removing aging nodes. Parameters are set as follows: η=0.6, μ=0.8, λ=0.5, δ=0.3, τ=0.2, γ=2, and κ=2. (**a**) p=0.9, q=0.05, r=0.05; (**b**) p=0.05, q=0.9, r=0.05; (**c**) p=0.05, q=0.05, r=0.9.

Second, in Figure 6, we draw $\rho^{R}$ as a function of $\beta_{1}$ for different dynamic parameters (p, q, r) and the lower layer mapping rate ϕ. When $\beta_{1}\in \left(0,1\right)$ is constant, it can be seen that the proportion of recovered enterprises increases with the lower layer mapping rate ϕ. There is no difference between Figure 6a,b, while the function image in Figure 6c decreases as a whole. This is because when the lower layer mapping rate is high, most enterprises will make wrong judgments after receiving uncertain information and then be infected by risks. In this case, the development of new cooperation and the transfer of partners will increase the probability of being infected, which will make risks greatly spread in the system. Only by eliminating outdated enterprises can the proportion of infected enterprises be reduced.

**Figure 7:**
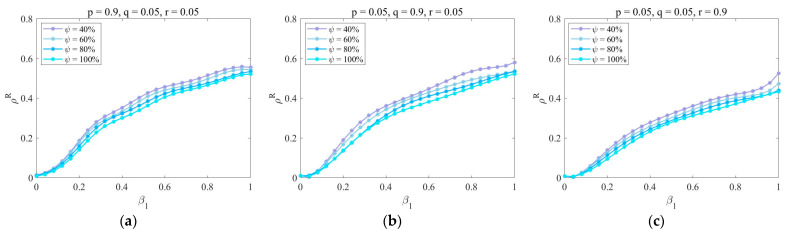
The impact of dynamic parameters (p, q, r) and the upper layer mapping rate ψ under the strategy of removing aging nodes. Parameters are set as follows: η=0.6, μ=0.8, λ=0.5, δ=0.3, τ=0.8, γ=2, and κ=2. (**a**) p=0.9, q=0.05, r=0.05; (**b**) p=0.05, q=0.9, r=0.05; (**c**) p=0.05, q=0.05, r=0.9.

Under different dynamic parameters (p, q, r), Figure 7 shows the change in $\rho^{R}$ with the increase in $\beta_{1}$ for different upper layer mapping rates ψ. Obviously, the proportion of recovered enterprises $\rho^{R}$ decreases with the increase in the upper mapping rate ψ. The function images in Figure 7a,b are basically the same, and the function image in Figure 7c moves down as a whole. Thus, too much cooperation is not suitable during the risk, and the elimination of outdated enterprises is conducive to market stability. The improvement of the upper layer mapping rate has increased the attention of enterprises to authoritative information, which will inhibit the spread of uncertain information and largely prevent the spread of risk.

### 4.2. Removal of Key Nodes

**Figure 8:**
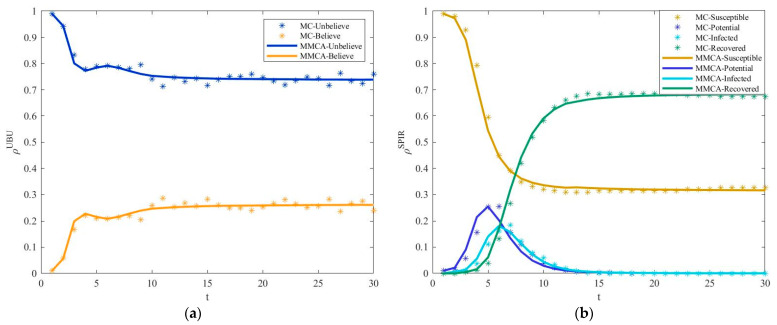
Under the strategy of removing key nodes. (**a**) The fraction of U-state and B-state enterprises as a function with time step t. (**b**) The fraction of S-state, P-state, I-state, and R-state enterprises as a function with time step *t*. Parameters are set as follows: p=0.5, q=0.45, β=0.5, η=0.6, μ=0.8, λ=0.5, δ=0.3, τ=0.8, γ=2, and κ=2.

It is the same as in Section 4.1. First, the effectiveness of MMCA in characterizing supply chain risk diffusion under the strategy of removing key nodes is verified. From Figure 8, it can be seen that the function image of the proportion of enterprises in U-, B-, S-, P-, I-, and R-states under MC simulation and MMCA iteration is basically consistent. For the proportion of U-state enterprises, the relative error is 1.60%. For the proportion of enterprises in the B-state, S-state, and R-state, the relative error is 5.11%, 3.07%, and 6.79%, respectively. Therefore, MMCA is suitable for continuing to test the impact of parameters on supply chain risk diffusion under the strategy of removing key nodes.

**Figure 9:**
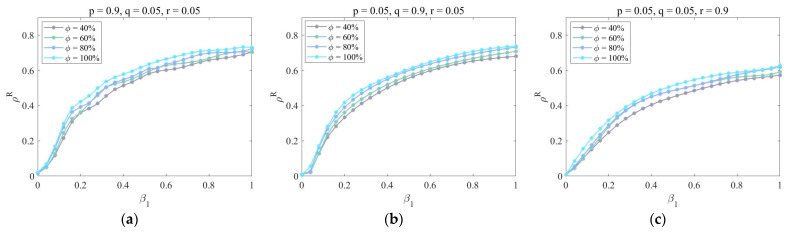
The impact of dynamic parameters (p, q, r) and the lower layer mapping rate ϕ under the strategy of removing key nodes. Parameters are set as follows: η=0.6, μ=0.8, λ=0.5, δ=0.3, τ=0.2, γ=2, and κ=2. (**a**) p=0.9, q=0.05, r=0.05; (**b**) p=0.05, q=0.9, r=0.05; (**c**) p=0.05, q=0.05, r=0.9.

Next, under different dynamic parameters (p, q, r), Figure 9 shows $\rho^{R}$ as a function of $\beta_{1}$ for different lower layer mapping rates ϕ. It is basically consistent with the results in Figure 6; $\rho^{R}$ increases with the upper layer mapping rate ϕ. Compared with Figure 9a,b, the function diagram of Figure 9c is significantly reduced. However, under the same parameter settings, the proportion of recovered enterprises in Figure 9 is larger than that in Figure 6 as a whole. This shows that under the strategy of removing key nodes, the large scale of risk diffusion leads to a large proportion of recovered enterprises. Although removing key nodes suppresses risks, there is still a certain gap between the effect and that of removing aging nodes.

**Figure 10:**
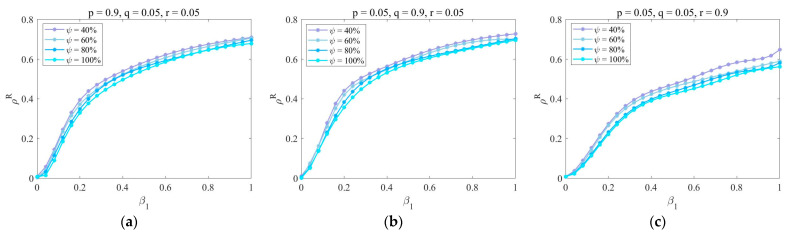
The impact of dynamic parameters (p, q, r) and the upper layer mapping rate ψ under the strategy of removing key nodes. Parameters are set as follows: η=0.6, μ=0.8, λ=0.5, δ=0.3, τ=0.8, γ=2, and κ=2. (**a**) p=0.9, q=0.05, r=0.05; (**b**) p=0.05, q=0.9, r=0.05; (**c**) p=0.05, q=0.05, r=0.9.

Finally, Figure 10 shows the impact of the upper layer mapping rate ψ on the proportion of recovered enterprises $\rho^{R}$ with the increase in $\beta_{1}$ under the different dynamic parameters (p, q, r). The results are consistent with those in Figure 7; the proportion of recovered enterprises $\rho^{R}$ decreases with the increase in the upper layer mapping rate ψ. The images in Figure 10a,b are basically the same, and the proportion of recovered enterprises in Figure 10c decreases as a whole. This once again proves that improving the upper layer mapping rate and removing key enterprises will curb risks, while it is not wise to cooperate during risk. Compared with Figure 7 with the same parameter settings, the proportion of recovered enterprises in Figure 10 increases as a whole. Therefore, in terms of curbing the diffusion of risks, it is indeed more effective to eliminate outdated enterprises than to eliminate key enterprises.

## 5. Conclusions

The key to supply chain management is to establish a strategic partnership, risk taking, and control. To better suppress risk diffusion in partially mapped double-layer hypernetworks, we consider two node removal strategies to establish a dynamic network evolution model. The node relationships between double-layer networks in the past were too idealized [26,29]. The inter-layer mappings in this paper are all randomly generated to make the model more realistic. Finally, numerical simulations are conducted to test the effects of the mapping rate and dynamic parameters on risk diffusion under different strategies, and the following major findings are obtained:

(1) The increase in the upper layer mapping rate and adding and removing nodes will inhibit the diffusion of risks.
(2) The increase in the lower layer mapping rate and adding and rewiring hyperedges will promote the risk diffusion.
(3) To restrain risk, it is more effective to remove aging nodes than key nodes.

The interlayer mapping affects the probability of being infected by risk. If the mapping exists, enterprises have social accounts and will make incorrect judgments when receiving uncertain information, increasing the probability of being infected. Without mapping, there is no impact. Based on the major findings above, enterprises with social accounts should focus on the authoritative information release of official media. The government should adjust the market structure in a timely manner, control key enterprises, and eliminate outdated enterprises. Regarding the advantages of the model, first, the partially mapping double-layer hypernetwork can better express the phenomenon that only some enterprises have social accounts, and only some social accounts follow official media in reality. Second, the contribution of two node removal strategies to risk suppression is compared, and a better risk suppression effect is obtained. Finally, this paper helps decision makers to better use internet information to establish cooperative relations and provides a theoretical basis for the government to administrate large-scale market risks. The disadvantage is that the paper only considers a single risk, while the actual risk is diverse. Furthermore, the cooperation between enterprises is also divided into intimacy and estrangement, and a type of hyperedge will overlook important information such as the degree of cooperation between different enterprises. Therefore, more efforts on building a propagation model and introducing a weighting matrix are expected in future works. The above provides inspiration for the study of supply chain risk diffusion behavior, supplier selection, contract design, and risk assessment.

## Figures and Tables

**Table 1:** Parameter and meaning.

Parameter	The Meaning of the Parameter
p	Probability of adding the hyperedge
q	Probability of rewiring the hyperedge
r	Probability of adding and removing nodes
N(t)	The total number of nodes
$\omega_{j}(t,t_{i})$	The probability of the jth node in the ith batch encountering risk
β	Probability of transition from the S-state to the P-state

## Data Availability

Not applicable.
